# Comparison of SP142 and 22C3 PD-L1 assays in a population-based cohort of triple-negative breast cancer patients in the context of their clinically established scoring algorithms

**DOI:** 10.1186/s13058-023-01724-2

**Published:** 2023-10-10

**Authors:** Gudbjörg Sigurjonsdottir, Tommaso De Marchi, Anna Ehinger, Johan Hartman, Ana Bosch, Johan Staaf, Fredrika Killander, Emma Niméus

**Affiliations:** 1 Division of Oncology, Department of Clinical Sciences Lund, Lund University, Lund, Sweden; 2 Department of Oncology and Radiation Physics, Skåne University Hospital, Lund, Sweden; 3 Department of Clinical Genetics, Pathology and Molecular Diagnostics, Laboratory Medicine, Region Skåne, Lund, Sweden; 4 Department of Oncology and Pathology, Karolinska Institute and University Hospital, Stockholm, Sweden; 5 Division of Translational Cancer Research, Department of Laboratory Medicine, Lund University, Medicon Village, Lund, Sweden; 6 Divison of Surgery, Department of Clinical Sciences Lund, Lund University, Sölvegatan 19 - BMC I12, 22184 Lund, Sweden; 7 Department of Surgery, Skåne University Hospital, Malmö, Sweden

**Keywords:** Triple-negative breast cancer, PD-L1, Immunohistochemistry, SP142, 22C3, Concordance, TILs, Patient outcome, Gene expression

## Abstract

**Background:**

Immunohistochemical (IHC) PD-L1 expression is commonly employed as predictive biomarker for checkpoint inhibitors in triple-negative breast cancer (TNBC). However, IHC evaluation methods are non-uniform and further studies are needed to optimize clinical utility.

**Methods:**

We compared the concordance, prognostic value and gene expression between PD-L1 IHC expression by SP142 immune cell (IC) score and 22C3 combined positive score (CPS; companion IHC diagnostic assays for atezolizumab and pembrolizumab, respectively) in a population-based cohort of 232 early-stage TNBC patients.

**Results:**

The expression rates of PD-L1 for SP142 IC ≥ 1%, 22C3 CPS ≥ 10, 22C3 CPS ≥ 1 and 22C3 IC ≥ 1% were 50.9%, 27.2%, 53.9% and 41.8%, respectively. The analytical concordance (kappa values) between SP142 IC+ and these three different 22C3 scorings were 73.7% (0.48, weak agreement), 81.5% (0.63) and 86.6% (0.73), respectively. The SP142 assay was better at identifying 22C3 positive tumors than the 22C3 assay was at detecting SP142 positive tumors. PD-L1 (*CD274*) gene expression (mRNA) showed a strong positive association with all two-categorical IHC scorings of the PD-L1 expression, irrespective of antibody and cut-off (Spearman Rho ranged from 0.59 to 0.62; all *p*-values < 0.001). PD-L1 IHC positivity and abundance of tumor infiltrating lymphocytes were of positive prognostic value in univariable regression analyses in patients treated with (neo)adjuvant chemotherapy, where it was strongest for 22C3 CPS ≥ 10 and distant relapse-free interval (HR = 0.18, *p* = 0.019). However, PD-L1 status was not independently prognostic when adjusting for abundance of tumor infiltrating lymphocytes in multivariable analyses.

**Conclusion:**

Our findings support that the SP142 and 22C3 IHC assays, with their respective clinically applied scoring algorithms, are not analytically equivalent where they identify partially non-overlapping subpopulations of TNBC patients and cannot be substituted with one another regarding PD-L1 detection.

*Trial registration* The Swedish Cancerome Analysis Network - Breast (SCAN-B) study, retrospectively registered 2nd Dec 2014 at ClinicalTrials.gov; ID NCT02306096.

**Supplementary Information:**

The online version contains supplementary material available at 10.1186/s13058-023-01724-2.

## Introduction

Patients with triple-negative breast cancer (TNBC) have poorer prognosis compared to patients with other breast cancer subtypes and fewer treatment options due to the absent or low expression of estrogen and progesterone receptors and HER2 [1]. Exploration of alternative therapy options for TNBC patients is ongoing and immune checkpoint inhibitors (ICIs) targeting the programmed death 1 (PD-1)/programmed death-ligand 1 (PD-L1) interaction are now approved in TNBC [2]. However, questions remain to be answered regarding the optimal selection of patients who might benefit from ICI treatment.

PD-L1 protein expression determined by immunohistochemistry (IHC) is currently the only clinically applied predictive biomarker for checkpoint inhibition in TNBC and is relevant in the unresectable locally advanced or metastatic setting. However, PD-L1 evaluation in breast cancer varies where each ICI comes with a companion/complementary IHC assay where the antibodies, scoring systems, definition of positivity and predictive threshold is different across assays [3–5]. For optimal clinical use of PD-L1 as a biomarker, a unique and harmonized IHC workflow and scoring system should be developed. Several phase III clinical trials with different ICIs in TNBC have shown mixed results, but some have been promising in the metastatic and neoadjuvant setting. Commonly investigated ICIs in TNBC are atezolizumab together with the SP142 Ventana IHC assay (IMpassion trials) and pembrolizumab together with the 22C3 Dako IHC assay (Keynote trials), and these ICIs have been approved in TNBC in combination with chemotherapy [6–19]. SP142 has been found to have less sensitivity for PD-L1 staining on tumor cells (TCs) than on immune cells (ICs) in TNBC and the scoring system for SP142 is based on the proportion of tumor area occupied by PD-L1 expressing ICs [20, 21]. For SP142, a predictive threshold value of 1% has been found when adding atezolizumab to nab-paclitaxel in metastatic TNBC in the IMpassion130 phase III trial that led to the first accelerated approval of an ICI in TNBC. The combination is approved outside of the US but has been withdrawn by FDA since continued approval was contingent upon the results of the IMpassion131 trial which failed at showing significant clinical benefit of atezolizumab in combination with paclitaxel [6, 11, 22]. On the other hand, the scoring system for the 22C3 antibody is a combined positive score (CPS) that is based on PD-L1 expression in TCs and ICs as a proportion of the total number of TCs. For 22C3, a predictive threshold value of CPS 10 has been found in the metastatic setting in the Keynote-355 phase III trial [7]. On the contrary, atezolizumab and pembrolizumab have in phase III trials shown clinical benefit in the neoadjuvant setting irrespective of PD-L1 status by SP142 and 22C3, respectively [9, 10, 14].

The IC+ scoring method is not clinically applied for 22C3, and CPS is not clinically applied for SP142. Several studies have shown inter-assay variability and discordance between the SP142 and 22C3 assays, each detecting partially non-overlapping subpopulations of PD-L1 positive TNBC patients [21, 23–29]. However, these studies have not been consistent in their comparison of scoring methods and their prognostic impacts. To our knowledge, only a few studies so far evaluated the agreement of the clinically established scoring algorithms of these assays in TNBC, reporting impaired concordance [21, 23, 25].

In our current study, we investigated the agreement between the SP142 and 22C3 assays in the context of their clinically used scoring systems in TNBC, assessed their correlation to PD-L1 expression at the mRNA level to investigate if the assays differ in their association with the mRNA status, and evaluated their prognostic value in a population-based early-stage TNBC cohort. The overall aim was to provide additional data about assay interchangeability to support PD-L1 analysis in TNBC and clinical decision making.

## Material and methods

### Patient cohort

**Fig. 1:**
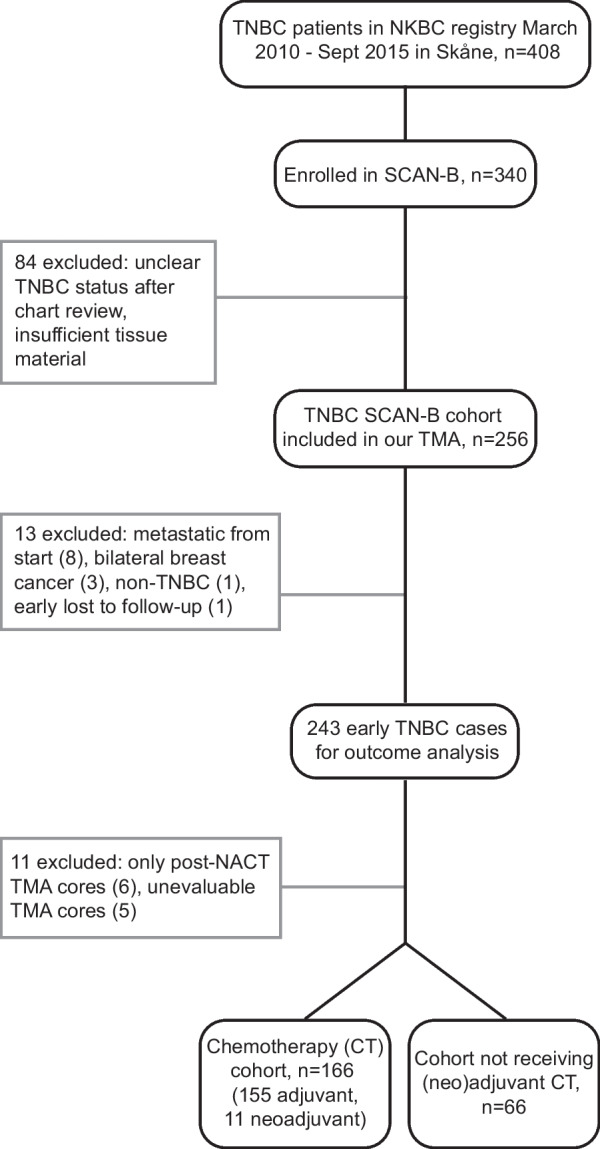
Study flowchart. Our final cohort consisted of 232 early-stage TNBC patients recruited from the population-based SCAN-B cohort. *Abbreviations* TNBC: triple-negative breast cancer; NKBC: National Breast Cancer Quality (NKBC) registry; SCAN-B: Swedish Cancerome Analysis Network - Breast; TMA: tissue microarray; NACT: neoadjuvant chemotherapy

The origin of our TNBC cohort has been previously described by Staaf et al. [30]. Briefly, a total of 408 TNBC patients were identified in Region Skåne between 2010/09 and 2015/03 by the Swedish National Breast Cancer Quality (NKBC) registry. Of those, 340 were enrolled in the Swedish Cancerome Analysis Network - Breast (SCAN-B) study (ClinicalTrials.gov ID NCT02306096), which is a population-based study in the southern health care region of Sweden and all patients with primary breast cancer are eligible (https://www.scan-b.lu.se/) [31]. Eighty-four patients were thereafter excluded because of unclear TNBC status or insufficient tissue material. Of the 256 remaining patients included in our tissue microarray (TMA), 13 were excluded due to metastatic disease at diagnosis or prior to start of adjuvant chemotherapy (n = 8), bilateral breast cancer (n = 3), loss to follow-up before treatment start (n = 1), or non-TNBC status (n = 1). Clinicopathological characteristics and follow-up data was collected through clinical chart review and the last date of counting in events was 18th Oct 2019. Additional 11 patients were excluded since they only had TMA cores from residual disease after neoadjuvant chemotherapy (n = 6) or due to unevaluable TMA cores for the 22C3 staining (n = 5). Of the remaining 232 patients, who all underwent primary surgery (mastectomy or partial mastectomy), 166 received chemotherapy (CT-cohort) according to national guidelines, of which 155 received adjuvant and 11 neoadjuvant CT. Of these, 98.2% (163 of 166) received FEC or EC (5-fluorouracil, epirubicin, cyclophosphamide) based treatment with or without a taxane and three patients (1.8%) received less than 50% of planned CT. The remaining 66 patients did not receive any neo(adjuvant) CT, most often due to age or comorbidities (non-CT-cohort). Checkpoint inhibitors were not given to the patients in the cohort. Adjuvant radiotherapy was given according to national guidelines. All of the 166 CT patients were eligible for overall survival (OS) analysis, 165 for invasive disease-free survival (IDFS) and 163 for distant relapse-free interval (DRFI). In the non-CT-cohort, 64 were eligible for OS, 65 for IDFS and 63 for DRFI (Fig. 1, study flowchart). Clinicopathological characteristics in the CT-cohort (prior to eventual (neo)adjuvant CT) and the non-CT-cohort are presented in Table 1. RNA sequencing data for gene expression profiling (GEX) was available for 84% of the patients (194 out of 232 patients) through the SCAN-B consortium [31].

**Table 1:** Clinicopathological characteristics

n (%)	Overall cohort N = 232	CT-cohort N = 166	Non-CT-cohort N = 66	*p*-value
*Age at diagnosis (years)*				
Median (range)	61 (26–91)	55.5 (26–76)	80 (38–91)	< 0.001
< 50	54 (23.3)	51 (30.7)	3 (4.5)	< 0.001
50–75	131 (56.5)	114 (68.7)	17 (25.8)	
≥ 76	47 (20.3)	1 (0.6)	46 (69.7)	
*Tumor size*				
≤ 20 mm	113 (48.7)	83 (50.0)	30 (45.5)	0.303
> 20 mm	107 (46.1)	71 (42.8)	36 (54.5)	
Unknown	12 (5.2)	12 (7.2)	0	
*Lymph node status*				
Node negative (N0)	145 (62.5)	100 (60.2)	45 (68.2)	0.228
Node positive (N +)	86 (37.1)	66 (39.8)	20 (30.3)	
Unknown	1 (0.4)	0	1 (1.5)	
*Nottingham histologic grade*				
1	0	0	0	0.004
2	30 (12.9)	14 (8.4)	16 (24.2)	
3	196 (84.5)	146 (88.0)	50 (75.8)	
Unknown	6 (2.6)	6 (3.6)	0	
*Ki-67 proliferation marker*				
≤ 30%	41 (17.7)	22 (13.3)	19 (28.8)	0.007
> 30%	189 (81.5)	143 (86.1)	46 (69.7)	
Unknown	2 (0.9)	1 (0.6)	1 (1.5)	
*Histological type*				
Invasive ductal carcinoma	183 (78.9)	132 (79.5)	51 (77.3)	0.004
Medullary features	16 (6.9)	16 (9.6)	0	
Other	33 (14.2)	18 (10.8)	15 (22.7)	
*TIL abundance, %*				
Median (range)	20 (0–100)	20 (0–90)	10 (0–100)	0.028
< 30%	139 (59.9)	94 (56.6)	45 (68.2)	0.100
≥ 30%	91 (39.2)	71 (42.8)	20 (30.3)	
Unknown	2 (0.9)	1 (0.6)	1 (1.5)	
*SP142 IC status*				
< 1%	114 (49.1)	72 (43.4)	42 (63.6)	0.006
≥ 1%	118 (50.9)	94 (56.6)	24 (36.4)	
*22C3 CPS status*				
< 1	107 (43.1)	68 (41.0)	39 (59.1)	0.028
≥ 1–9	62 (26.7)	51 (30.7)	11 (16.7)	
≥ 10	63 (27.2)	47 (28.3)	16 (24.2)	
*22C3 IC status*				
< 1%	135 (58.2)	91 (54.8)	44 (66.7)	0.107
≥ 1%	97 (41.8)	75 (45.2)	22 (33.3)	
*Follow-up time*				
Median, months (range)	68 (5–108)	70 (5–108)	64 (6–106)	0.014
< 2 y	20 (8.6)	9 (5.4)	11 (16.7)	0.009
2–5 y	51 (22.0)	35 (21.1)	16 (24.2)	
> 5 y	159 (68.5)	122 (73.5)	37 (56.1)	
Unknown	2 (0.9)	0	2	
*Events, yes event occured*				
Death (OS event)	54 (23.3)	33 (19.9)	21 (31.8)	0.059
Relapse (distant or locoregional)	49 (21.1)	34 (20.5)	15 (22.7)	0.720
Relapse or death (IDFS event)	69 (29.7)	42 (25.3)	27 (40.9)	0.026
Distant relapse (DRFI event)	39 (16.8)	28 (16.9)	11 (16.7)	1.000

### PD-L1 immunohistochemistry (IHC) and tissue microarray (TMA)

Scoring of PD-L1 expression by immunohistochemical testing was assessed in formalin-fixed, paraffin-embedded tumor samples in a TMA, using two different PD-L1 antibody clones: SP142 with Ventana BenchMark Ultra platform (Ventana Medical Systems, Inc., AZ, U.S) and 22C3 with Dako Autostainer Link 48 platform (Agilent, Inc., CA, U.S) IHC assays. Preparation and staining were done according to the manufacturer´s instructions. The TMA images were assessed in PathXL Philips Xplore (Koninklijke Philips N.V., NL). Each sample was represented by two TMA cores, each of 1.0 mm in diameter. PD-L1 in the adjuvant treated patients and the non-CT-cohort was evaluated on TMA cores from the surgical specimen. For the neoadjuvant patients, PD-L1 was evaluated on TMA cores from core needle biopsies taken prior to neoadjuvant treatment.

### PD-L1 IHC scoring

**Fig. 2:**
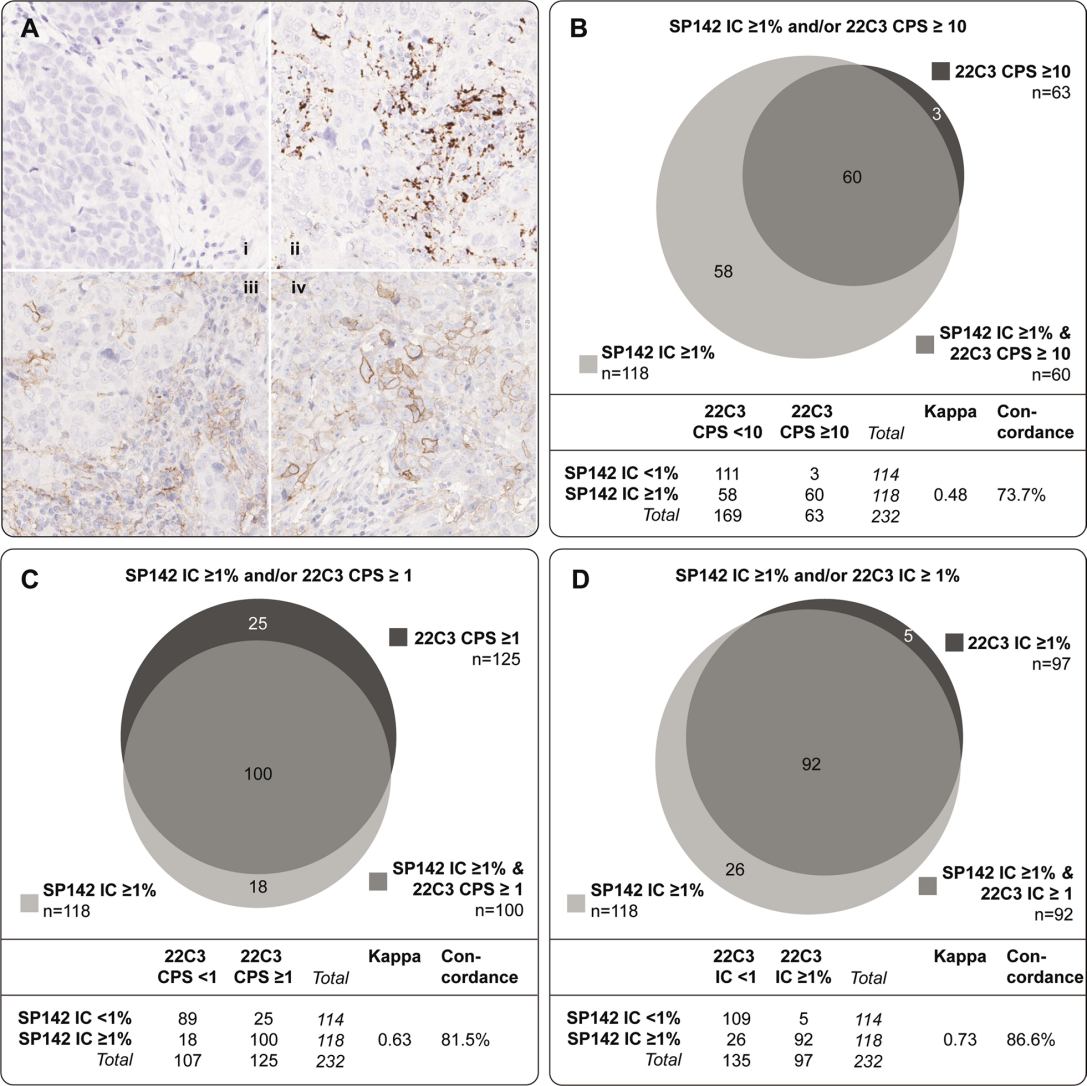
Tissue microarray immunohistochemical (IHC) images of PD-L1 staining and comparison of assays. **A**:i Negative PD-L1 staining with SP142. **A**:ii Positive PD-L1 staining in immune cells (ICs) with the SP142 antibody. **A**:iii Positive 22C3 staining, mostly in ICs. ii and iii are from the same tumor. **A**:iv Positive PD-L1 staining in tumor cells and in ICs with the 22C3 antibody. All images at 20 × magnification. Concordance analyses in the overall cohort (N = 232) between the SP142 and 22C3 assays with different scoring algorithms where SP142 IC ≥ 1% is compared to 22C3 combined positive score (CPS) ≥ 10, 22C3 CPS ≥ 1 and to 22C3 IC ≥ 1% in (**B**–**D**), respectively. Venn diagrams show the overlap between the assay IHC expressions, kappa values represent the measurement of the level of agreement and the concordance rate equals the overall percentage agreement

We evaluated PD-L1 staining according to two scoring methods: CPS and staining in ICs. CPS was defined as the combined number of PD-L1 stained TCs, tumor infiltrating lymphocytes (TILs) and macrophages (intratumorally and in adjacent stroma) divided by the total number of TCs, multiplied by 100. We evaluated CPS at a threshold of 1 and 10 according to PD-L1 evaluation in clinical phase III TNBC studies with pembrolizumab and the 22C3 assay [7, 8, 10]. The IC+ score was defined as percentage of the tumor area (non-necrotic, non-sclerotic area) covered by PD-L1 stained tumor infiltrating ICs and evaluated at a threshold of 1% as performed in phase III TNBC trials with atezolizumab and the SP142 assay [6, 9, 11, 13]. The score from the TMA core with highest value was set as the respective CPS and IC+ score for the tumor. PD-L1 expression in TCs (in CPS) included partial or complete membranous staining and in ICs (in CPS and IC+) membranous and/or cytoplasmic staining. Scoring of SP142 PD-L1 expression was done by a physician and a board-certified breast cancer pathologist where consensus in non-matching scoring had to be reached for 4,7% of the tumors (Additional file 1: Table S1). The 22C3 scoring was performed by a physician and in cases that were not clearly obvious, a board-certified breast cancer pathologist was consulted and consensus reached. IHC staining examples are illustrated in Fig. 2A. We scored CPS using the 22C3 assay and IC+ with both SP142 and 22C3 (note it is experimental scoring of IC+ with 22C3 since the IC+ scoring is not clinically applied for 22C3). We did not evaluate CPS for SP142 since it has been shown to have impaired sensitivity for PD-L1 staining in TCs in TNBC [20, 21]. When investigating the concordance between the assays, the SP142 IC+ of ≥ 1% and 22C3 CPS of ≥ 10 scores were compared as they are the only clinically established predictive cut-offs in TNBC. Moreover, since 22C3 CPS ≥ 1 also has been investigated in clinical trials, the concordance between SP142 IC+  ≥ 1% and 22C3 CPS ≥ 1 was evaluated. In addition to this, to compare under more similar, but explorative, scoring conditions, the concordance between SP142 IC+ and 22C3 IC+ was evaluated.

### Evaluation of tumor infiltrating lymphocytes (TILs)

Abundance of stromal TILs was evaluated by a board-certified breast cancer pathologist on hematoxylin–eosin stained whole slides from surgical specimen before eventual adjuvant chemotherapy and from pre-treatment core needle biopsies for the neoadjuvant treated patients. Abundance was calculated as percentage of TILs occupying the tumoral stromal area according to the international TILs working group (https://www.tilsinbreastcancer.org/) [32]. If more than one slide was available per patient, the average score was applied. Threshold for high versus low TILs (as binary variable) was set to 30% as performed in a previous pooled analysis of the prognostic value of TILs in early-stage TNBC patients [33], which also was near the mean value of TIL abundance in our cohort (27% in the overall cohort, 29% in the CT-cohort).

### Clinical endpoints

In the survival analyses, OS, IDFS and DRFI were defined as endpoints with support of the STEEP criteria [34]. OS was the time from diagnosis of primary breast cancer to death of any cause. IDFS was the time from primary diagnosis to the diagnosis of a breast cancer related invasive event (locoregional or distant) or, if no relapse had occurred, to death of any cause. In the absence of event in OS and IDFS the case was censored at last follow-up. DRFI was defined as the time from diagnosis to the diagnosis of a distant relapse of breast cancer or breast cancer related death, the case was censored at death of any other cause or at last follow-up if no DRFI event had occurred. Contralateral breast cancer and distant recurrences with uncertain origin were not included in DRFI but were included in IDFS. Follow-up time was defined as the time from diagnosis to date of death or to last follow-up.

### Statistical analyses and analyses of RNA sequencing data

Analyses of RNAseq data were performed in R (v 3.6.1), all remaining statistical analyses with SPSS (v 26.0). Concordance rate (expressed as percentage) was calculated to evaluate IHC inter-test reliability and kappa statistic applied as a measurement of the level of agreement. A kappa coefficient of ≥ 0.80 was interpreted as strong agreement, 0.60–0.79 as good, 0.40–0.59 as weak, 0.21–0.39 as minimal and < 0.20 as none agreement [35]. Area-proportional Venn diagrams were drawn with https://www.biovenn.nl/ [36]. Chi-square test was applied when comparing categorical values between groups (chi-square test for trend if more than two groups were compared). Nonparametric Mann–Whitney test was applied to compare non-categorical values between two groups. Survival data were analyzed by Kaplan–Meier estimates along with log-rank test and with Cox regression, reporting hazard ratio (HRs) and 95% confidence intervals (CIs). Multivariable Cox regression analyses were performed by including, aside from PD-L1 status, other traditional and prognostic factors: age at diagnosis, tumor size, lymph node status, Nottingham histologic grade (NHG) and TIL abundance as binary covariates. Four multivariable regression analyses were performed, i.e., one for each PD-L1 scoring method: SP142 IC+ , 22C3 CPS 10, 22C3 CPS 1 and 22C3 IC+. RNA sequencing data was matched against patient data generating a list of 16,258 genes across 194 samples. FKPM values were Log2-transformed, imputed (missing data to 0), mean-centered and scaled (samples and genes). The correlation between PD-L1 gene expression (by RNAseq) and PD-L1 protein expression (analyzed by IHC) was estimated using the Spearman method and visualized with boxplots (the median is indicated by the central line, upper and lower limits of the box represent the upper and lower quartiles and whiskers the × 1.5 interquartile range). A *p*-value less than 0.05 was considered statistically significant and all *p*-tests were two sided.

## Results

### Frequency of PD-L1 IHC expression

A higher positive detection rate (Table 1) was observed for SP142 IC ≥ 1% than for 22C3 CPS ≥ 10, 50.9% (118/232) versus 27.2% (63/232), when using these clinically applied predictive cut-offs (from the advanced TNBC setting).

Since 22C3 CPS ≥ 1 has also been investigated in clinical trials, we analyzed the percentage of PD-L1 positivity using this lower cut-off for 22C3. As expected, this resulted in a higher positive detection rate (53.9% (125/232)) compared to 22C3 CPS ≥ 10.

In an explorative analysis, to evaluate 22C3 under more similar conditions as SP142, we applied the IC+ scoring method to 22C3. The positive detection rate for 22C3 IC ≥ 1% was 41.8% (97/232).

### Comparison between SP142 and 22C3

When comparing SP142 and 22C3 with the clinically applied scoring methods and cut-offs (IC ≥ 1% and CPS ≥ 10, respectively), a kappa value of 0.48 was obtained (interpreted as week agreement). Approximately half of the tumors (47.8%; 111/232) were negative with both antibodies, whereas 60 tumors (25.9%) were positive with both antibodies, resulting in a concordance rate of 73.7%. Fifty-eight tumors (25%) were positive with SP142, but negative with 22C3, whereas three tumors (1.3%) showed the opposite pattern (Fig. 2B). Taken together, almost half of the tumors (49.2%; 58/118) that stained PD-L1 positive with SP142 were considered to be negative with 22C3, when using these clinically established predictive cut-offs.

The kappa value increased to 0.63 (interpreted as good agreement) and the concordance rate to 81.5% when a threshold of ≥ 1 for CPS was applied for 22C3 where 189 tumors (out of 232) showed concordant PD-L1 status (89 tumors negative with both antibodies and 100 tumors positive with both; Fig. 2C). A lower number of tumors that stained positive with SP142 but negative with 22C3 was found than when using the ≥ 10 cut-off for CPS (n = 18 vs. n = 58). The number of tumors with the opposite pattern (i.e. negative with SP142 but positive with 22C3) was increased from 3 to 25.

Next, we evaluated the concordance between the two antibodies when scored with the same scoring method and cut-off, i.e. IC ≥ 1% (Fig. 2D, note that IC+ is not normally employed for the 22C3 antibody). This comparison resulted in the best concordance rate of 86.6% (201 concordant tumors: 109 negative with both and 92 positive with both) and a kappa-value of 0.73 (interpreted as good agreement). Five tumors were negative with SP142 but positive with 22C3 and 26 showed the opposite pattern.

### Association of SP142 and 22C3 with PD-L1 (CD274) gene expression (mRNA)

**Fig. 3:**
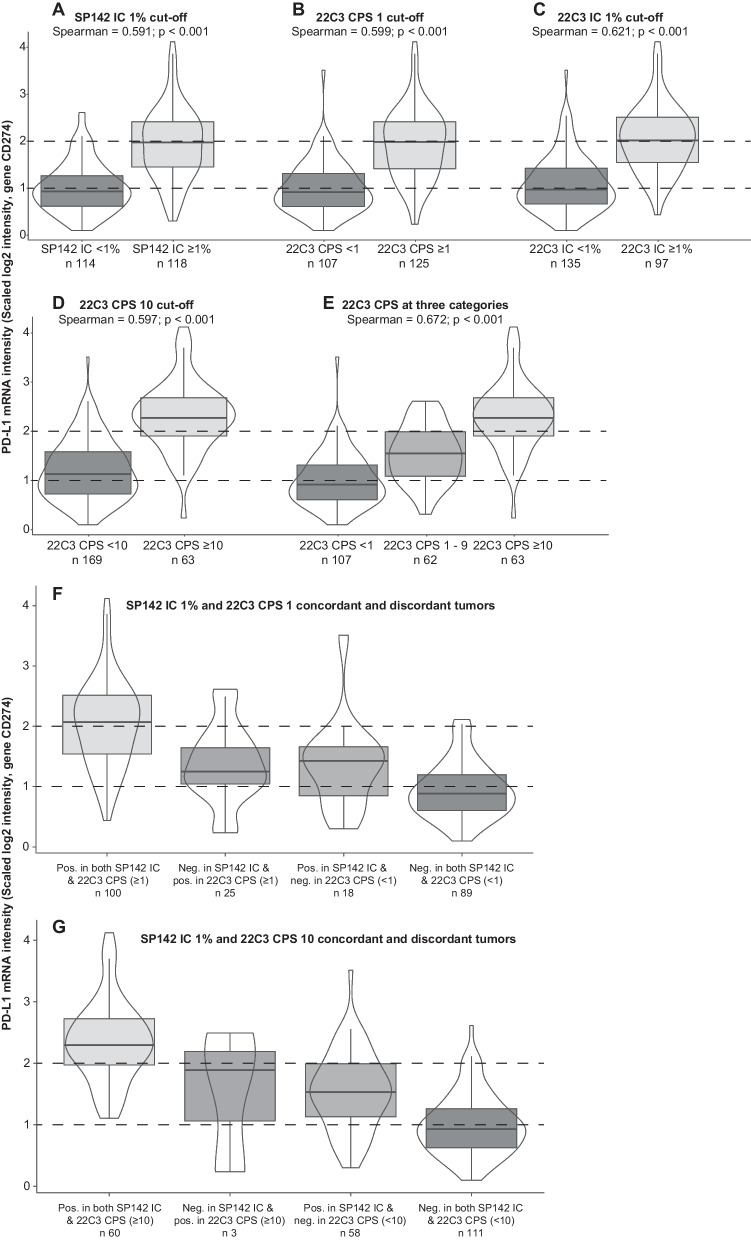
Association of immunohistochemical (IHC) PD-L1 expression with PD-L1 (*CD274*) gene expression (mRNA) in the overall cohort. In **A**–**D** the association of gene expression with SP142 PD-L1 staining in immune cells (ICs), 22C3 combined positive score (CPS) ≥ 1, 22C3 IC staining and 22C3 CPS ≥ 10, respectively, all at two-categorical IHC expressions. In **E** the association of gene expression with 22C3 CPS at three-categorical IHC expression. The mRNA expression of the SP142 IC and 22C3 CPS concordant and discordant cases in (**F**) and (**G**), with CPS threshold of 1 and 10, respectively

We detected a significant positive association between PD-L1 IHC expression and PD-L1 (*CD274*) gene expression (mRNA) in the overall cohort. The Spearman correlation coefficients were similar between PD-L1 gene expression and all the two-categorical IHC scorings (r_s_ = 0.59 for SP142 IC+, r_s_ = 0.60 for both 22C3 CPS 1 and CPS 10, r_s_ = 0.62 for 22C3 IC+; all *p*-values < 0.001; Fig. 3A–D). When stratifying the 22C3 CPS into three categories (i.e. < 1, 1–9 and ≥ 10), a positive stepwise association between PD-L1 (*CD274*) gene expression and PD-L1 protein expression was observed (Fig. 3E; r_s_ = 0.67), establishing a good degree of association between transcript and protein measurements.

We also investigated PD-L1 gene expression levels in SP142 IC and 22C3 CPS concordant and discordant groups, respectively (Fig. 3F, G). Here, transcript levels in the discordant groups (i.e. 22C3 CPS < 10 and SP142 IC ≥ 1% or 22C3 CPS ≥ 10 and SP142 IC < 1%) were found to be at an intermediate level between the concordant positive group and the concordant negative group. No significant difference in PD-L1 mRNA expression was found between the two discordant groups.

### Clinicopathological features in the CT-cohort and the non-CT-cohort

Clinicopathological characteristics differed in patients receiving (neo)adjuvant CT and in those not receiving CT. The patients in the CT-cohort were younger (*p* < 0.001), had higher median TIL abundance, more proliferative (*p* = 0.007) and higher-grade tumors (*p* = 0.004), higher rate of PD-L1 expressing tumors (*p* = 0.006 for SP142 IC and *p* = 0.028 for 22C3 CPS status) and tended to have fewer deaths (*p* = 0.059) but had similar rate of relapses as compared to the non-CT-cohort (Table 1). Due to these differences, we chose to evaluate clinicopathological features in relation to PD-L1 status and perform outcome analyses separately in the CT-cohort and the non-CT-cohort.

### Association of PD-L1 status with clinicopathological features

In the CT-cohort, tumors with SP142 IC ≥ 1% were significantly associated with higher NHG (*p* = 0.004), higher Ki-67 proliferation index (*p* = 0.005), histological medullary features (*p* = 0.001) and increased stromal TIL abundance (*p* < 0.001), whereas age at diagnosis, tumor size and lymph node status were not significantly associated with PD-L1 status (Table 2). When using CPS ≥ 10 for 22C3, only medullary features and TIL abundance were significantly associated with PD-L1 status (both *p* values < 0.001) and the association between PD-L1 and NHG and Ki-67 did not reach statistical significance (Table 2). With the other cut-offs for 22C3 (CPS ≥ 1 and IC ≥ 1%; Additional file 2: Table S2), the results were similar to those obtained for SP142, with significant associations to NHG (*p* < 0.001 and *p* = 0.012, respectively), Ki-67 level (*p* = 0.009 and *p* = 0.006, respectively), medullary features (*p* = 0.002 for both 22C3 CPS 1 and 22C3 IC+) and TIL abundance (*p* < 0.001 for both CPS 1 and IC+).

**Table 2:** Clinicopathological features in the CT-cohort in relation to SP142 and 22C3 CPS 10 PD-L1 status

n (%)	SP142 IC 1% cut-off			22C3 CPS 10 cut-off		
	IC < 1% N = 72 (43.4%)	IC ≥ 1% N = 94 (56.6%)	*p*-value	CPS < 10 N = 119 (71.7%)	CPS ≥ 10 N = 47 (28.3%)	*p*-value
*Age at diagnosis, years*						
Median (range)	58 (26–74)	54 (28–76)	0.237	56 (26–76)	54 (31–75)	0.851
< 50 y	21 (29.2)	30 (31.9)	0.737	36 (30.3)	15 (31.9)	0.853
≥ 50 y	51 (70.8)	64 (68.1)		83 (69.7)	32 (68.1)	
*Tumor size*						
≤ 20 mm	32 (44.4)	51 (54.3)	0.869	54 (45.4)	29 (61.7)	0.222
> 20 mm	29 (40.3)	42 (44.7)		53 (44.5)	18 (38.3)	
Unknown	11 (15.3)	1 (1.1)		12 (10.1)	0	
*Lymph node status*						
N0	40 (55.6)	58 (61.7)	0.749	71 (59.7)	29 (61.7)	0.862
N +	32 (44.4)	36 (38.3)		48 (40.3)	18 (38.3)	
*Histologic grade*						
1	0	0		0	0	
2	11 (15.3)	3 (3.2)	0.004	13 (10.9)	1 (2.1)	0.067
3	55 (76.4)	91 (96.8)		100 (84.0)	46 (97.9)	
Unknown	6 (8.3)	0		6 (5.0)	0	
*Ki-67*						
≤ 30%	16 (22.2)	6 (6.4)	0.005	18 (15.1)	4 (8.5)	0.316
> 30%	55 (76.4)	88 (93.6)		100 (84.0)	43 (91.5)	
Unknown	1 (1.4)	0		1 (0.8)	0	
*Histological type*						
Invasive ductal carcinoma	59 (81.9)	73 (77.7)	0.001	98 (82.4)	34 (72.3)	< 0.001
Medullary features	1 (1.4)	15 (16.0)		5 (4.2)	11 (23.4)	
Other	12 (16.7)	6 (6.4)		16 (13.4)	2 (4.3)	
*TIL abundance*						
Median, % (range)	10 (0–60)	40 (1–90)	< 0.001	15 (0–80)	50 (5–90)	< 0.001
< 30%	58 (80.6)	36 (38.3)	< 0.001	84 (70.6)	10 (21.3)	< 0.001
≥ 30%	14 (19.4)	57 (60.6)		34 (28.6)	37 (78.7)	
Unknown	0	1 (1.1)		1 (0.8)	0	

In the non-CT-cohort, a significant positive association between TIL abundance and PD-L1 status was observed, irrespective of PD-L1 IHC evaluation method (all *p*-values < 0.001 for SP142 IC+ , 22C3 CPS 1 and 22C3 IC+ ; for 22C3 CPS 10: *p* = 0.006 for median TIL score and *p* = 0.026 for TILs as binary covariate). Associations between the other clinicopathological parameters and SP142 IC ≥ 1%, 22C3 CPS ≥ 10 or 22C3 IC ≥ 1% did not reach significance (Additional file 3: Table S3). When using 22C3 CPS ≥ 1 cut-off, NHG was significantly associated with PD-L1 IHC expression (*p* = 0.045) and Ki-67 borderline significant (*p* = 0.051; Additional file 3: Table S3).

### Association of PD-L1 with patient outcome in the CT-cohort

**Fig. 4:**
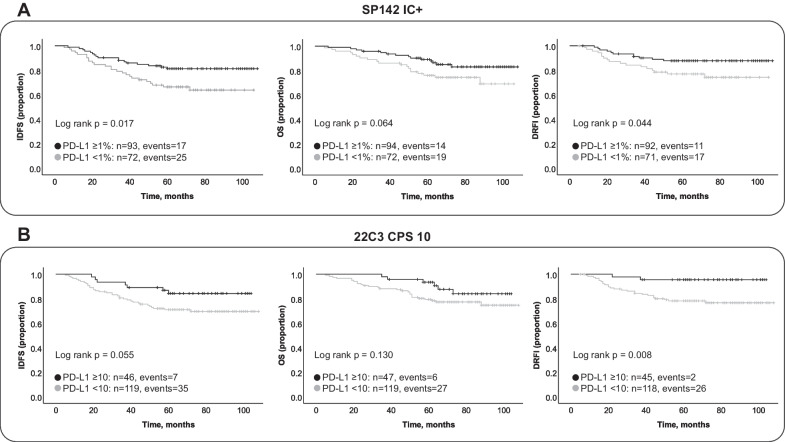
Kaplan Meier survival analyses according to immunohistochemical PD-L1 status in the cohort receiving (neo)adjuvant chemotherapy. Invasive disease-free survival (IDFS), overall survival (OS) and distant relapse-free interval (DRFI) according to SP142 PD-L1 expression in immune cells (IC+) in panel (**A**) and in panel (**B**) for 22C3 combined positive score (CPS) at a threshold of 10

When using the clinically established cut-offs for both SP142 (IC ≥ 1%) and 22C3 (CPS ≥ 10) in univariable Cox regression analyses, a positive PD-L1 status was significantly associated with a better DRFI (HR = 0.47, 95% CI 0.22–1.00, *p* = 0.049 for SP142 IC+ and HR = 0.18, 95% CI 0.04–0.76, *p* = 0.019 for 22C3 CPS 10; Table 3 and Fig. 4). The HRs for IDFS and OS also indicated a better prognosis for patients with PD-L1 positive tumors (HRs ranging from 0.46 to 0.53), but only reaching significant level for IDFS and SP142 IC status (95% CI 0.26–0.89, *p* = 0.02). The results for 22C3 CPS ≥ 1 and 22C3 IC ≥ 1% showed a similar pattern although only reaching significancy for 22C3 CPS 1 and IDFS (HR = 0.53, 95% CI 0.29–0.98, *p* = 0.043; Table 3 and Additional file 3: Fig. S1).

**Table 3:** Univariable and multivariable regression analyses in the CT-cohort (N = 166)

	IDFS univariable (events = 42)		IDFS multivariable (events = 37)		OS univariable (events = 33)		OS multivariable (events = 29)		DRFI univariable (events = 28)		DRFI multivariable (events = 23)	
	HR (95% CI)	*p*	HR (95% CI)	*p*	HR (95% CI)	*p*	HR (95% CI)	*p*	HR (95% CI)	*p*	HR (95% CI)	*p*
*(A)*												
Age	1.22 (0.61–2.42)	0.572	0.86 (0.39–1.92)	0.716	1.60 (0.70–3.70)	0.268	1.65 (0.60–4.49)	0.330	0.92 (0.42–2.04)	0.845	0.77 (0.30–1.98)	0.593
Tumor size	0.84 (0.43–1.61)	0.596	0.59 (0.29–1.21)	0.151	1.35 (0.65–2.79)	0.425	1.17 (0.54–2.55)	0.690	1.36 (0.60–3.08)	0.463	0.93 (0.39–2.25)	0.878
Lymph nodes	1.58 (0.86–2.90)	0.138	1.73 (0.86–3.49)	0.127	1.51 (0.76–2.99)	0.236	1.29 (0.59–2.82)	0.518	2.34 (1.11–4.95)	0.026	2.00 (0.84–4.80)	0.118
Histologic grade	0.68 (0.27–1.75)	0.427	1.01 (0.37–2.72)	0.992	0.51 (0.21–1.70)	0.330	0.91 (0.30–2.74)	0.863	1.09 (0.26–4.62)	0.909	1.29 (0.29–5.83)	0.739
TIL abundance	0.38 (0.19–0.78)	0.008	0.27 (0.11–0.67)	0.005	0.47 (0.22–1.01)	0.053	0.49 (0.19–1.28)	0.146	0.50 (0.22–1.13)	0.095	0.33 (0.11–0.99)	0.047
SP142 IC < 1% versus ≥ 1%	0.48 (0.26–0.89)	0.020	0.76 (.38–1.54)	0.450	0.53 (0.26–1.05)	0.069	0.78 (0.35–1.75)	0.544	0.47 (0.22–1.00)	0.049	0.80 (0.33–1.93)	0.612
*(B)*												
Age			0.84 (0.38–1.87)	0.675			1.61 (0.59–4.38)	0.348			0.78 (0.31–2.00)	0.610
Tumor size			0.60 (0.30–1.21)	0.155			1.16 (0.53–2.53)	0.704			0.91 (0.38–2.19)	0.840
Lymph nodes			1.69 (0.84–3.39)	0.139			1.28 (0.59–2.80)	0.531			2.11 (0.89–5.01)	0.090
Histologic grade			0.95 (0.36–2.54)	0.920			0.87 (0.29–2.60)	0.805			1.39 (0.31–6.16)	0.664
TIL abundance			0.27 (0.11–0.68)	0.006			0.48 (0.18–1.30)	0.147			0.47 (0.16–1.39)	0.173
22C3 CPS < 10 versus ≥ 10	0.46 (0.21–1.04)	0.061	0.82 (0.33–2.04)	0.666	0.51 (0.21–1.24)	0.137	0.83 (0.30–2.34)	0.728	0.18 (0.04–0.76)	0.019	0.26 (0.06–1.20)	0.084
*(C)*												
Age			0.84 (0.38–1.88)	0.677			1.63 (0.60–4.42)	0.342			0.77 (0.30–1.97)	0.588
Tumor size			0.61 (0.30–1.23)	0.167			1.19 (0.55–2.60)	0.658			0.96 (0.40–2.30)	0.926
Lymph nodes			1.67 (0.83–3.33)	0.149			1.27 (0.58–2.74)	0.550			1.95 (0.82–4.62)	0.131
Histologic grade			0.97 (0.36–2.63)	0.949			0.90 (0.30–2.74)	0.859			1.39 (0.31–6.27)	0.668
TIL abundance			0.27 (0.10–0.70)	0.007			0.51 (0.18–1.42)	0.198			0.41 (0.12–1.34)	0.138
22C3 CPS < 1 versus ≥ 1	0.53 (0.29–0.98)	0.043	0.86 (0.40–1.81)	0.684	0.56 (0.28–1.10)	0.093	0.79 (0.33–1.90)	0.595	0.49 (0.23–1.04)	0.063	0.59 (0.22–1.57)	0.293
*(D)*												
Age			0.84 (0.38–1.86)	0.658			1.61 (0.59–4.37)	0.359			0.77 (0.30–1.97)	0.588
Tumor size			0.60 (0.30–1.22)	0.159			1.17 (0.54–2.60)	0.688			0.96 (0.40–2.31)	0.924
Lymph nodes			1.67 (0.83–3.33)	0.150			1.26 (0.58–2.74)	0.558			1.97 (0.83–4.70)	0.125
Histologic grade			0.92 (0.34–2.45)	0.861			0.85 (0.29–2.54)	0.773			1.28 (0.29–5.70)	0.748
TIL abundance			0.24 (0.09–0.63)	0.003			0.44 (0.16–1.22)	0.116			0.37 (0.11–1.16)	0.088
22C3 IC < 1% versus ≥ 1%	0.57 (0.30–1.08)	0.086	1.03 (0.48–2.21)	0.936	0.66 (0.33–1.35)	0.256	1.00 (0.42–2.38)	0.992	0.47 (0.21–1.06)	0.068	0.70 (0.26–1.91)	0.488

Age at diagnosis: < 50 y (ref.) versus ≥ 50 y. Tumor size: ≤ 20 mm (ref.) versus > 20 mm. Lymph node status: node negative (ref.) versus node positive

Nottingham histologic grade: grade 2 (ref.) versus grade 3. TIL abundance: < 30% (ref.) versus ≥ 30%

Spaces left empty for univariable results in (B), (C) and (D) since the values are the same as in (A)

**Fig. 5:**
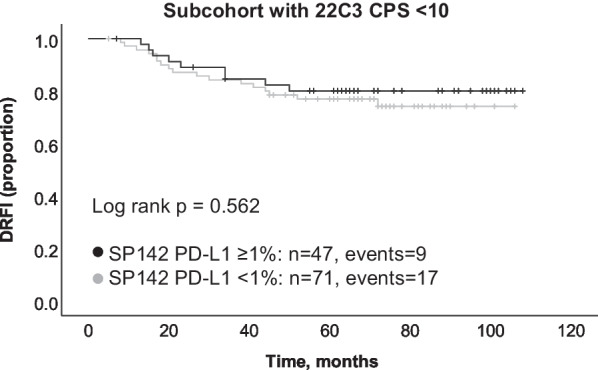
Subgroup survival analysis of patients with 22C3 combined positive score (CPS) < 10. Kaplan Meier estimates and log rank *p*-value for distant relapse-free interval (DRFI) according to SP142 PD-L1 status in the (neo)adjuvant chemotherapy sub-cohort that had 22C3 CPS < 10 (N = 119)

Next, we performed a subgroup analysis where we divided the 22C3 CPS 10 negative group (i.e., those with CPS < 10) into one group positive with SP142 (i.e. IC ≥ 1%; n = 47) and one group negative with SP142 (IC < 1%, n = 71). No significant difference in DRFI was observed between these two groups (log rank *p* = 0.562; Fig. 5). For the group with 22C3 CPS ≥ 10, a similar division was not meaningful since all the patients in the CT-cohort that had 22C3 CPS ≥ 10 also scored SP142 IC ≥ 1%. These results suggest that if information for PD-L1 status with 22C3 CPS 10 is available, SP142 does not add any further prognostic information for DRFI.

In multivariable Cox regression analysis, PD-L1 status was found not significantly associated to outcome for any of the clinical endpoints, irrespective of IHC assay and cut-off (Table 3). Of note though, a trend towards better DRFI was observed for 22C3 CPS ≥ 10 staining (HR = 0.26, 95% CI 0.06–1.20, *p* = 0.084). Stromal TIL abundance was the only covariate showing independent significant association to outcome in multivariable analyses, where it was positively associated with improved IDFS irrespective of PD-L1 assay and cut-off included in the analysis (HRs ranging from 0.24 to 0.27 and *p*-values from 0.003 to 0.007, Table 3A-D) and with a better DRFI in a multivariable model where SP142 IC+ was included (HR = 0.33, 95% CI 0.11–0.99, *p* = 0.047; Table 3A).

### Association of PD-L1 with patient outcome in the non-CT-cohort

The scarcity of patients in the non-CT-cohort did not allow for robust multivariable Cox regression analyses. PD-L1 status was not significantly associated with DRFI in univariable analysis (HRs ranging from 0.56 to 0.77, *p*-values not significant). For IDFS, the HRs for PD-L1 status were similar as in the CT-cohort (HRs ranging from 0.53 to 0.65 compared 0.46 to 0.57 in the CT-cohort), but in this small group of TNBC patients not treated with (neo)adjuvant CT with few events, the *p*-values were not significant (Additional file 5: Table S4 and Additional file 6: Fig. S2). Stromal TIL abundance was not significantly associated with any of the clinical endpoints in univariable analyses (HRs ranging from 0.90 to 1.34). Age (as continuous variable) was negatively associated with OS (HR = 1.07, 95% CI 1.01–1.14, *p* = 0.027) and IDFS (HR = 1.05, 95% CI 1.00–1.11, *p* = 0.043), tumor size was negatively associated with all the endpoints (HR = 2.41, 95% CI 1.08–5.39, *p* = 0.003 for IDFS; HR = 2.54, 95% CI 1.02–6.32, *p* = 0.045 for OS; HR = 4.63, 95% CI 1.00–21.53, *p* = 0.051 for DRFI) and lymph node status negatively associated with DRFI (HR = 8.06, 95% CI 2.13–30.59, *p* = 0.002; Additional file 5: Table S4).

## Discussion

To date, two different immune checkpoint inhibitors (ICIs) have been incorporated in the treatment of TNBC; pembrolizumab in both early-stage and metastatic TNBC and atezolizumab in the metastatic setting. Atezolizumab is still approved outside of the US but has been withdrawn by the FDA for metastatic TNBC. Each of these ICIs comes with a different PD-L1 IHC antibody assay, Ventana SP142 and Dako 22C3, respectively, that have different scoring methods and cut-offs [17, 18]. It is of clinical interest to harmonize these assays in the attempt to simplify the use of PD-L1 IHC expression as a predictive biomarker for checkpoint inhibition response. In this context, it has been recommended that a concordance rate of at least 90% is needed for assays to be considered analytically equivalent [37]. In our analysis, the comparison between SP142 IC ≥ 1% and 22C3 CPS ≥ 10, the currently clinically applied scoring methods and predictive cut-offs, showed a concordance rate of only 73.7% and kappa value of 0.48. These results indicate a weak concordance, as previously reported [21, 23]. This low rate of concordance in our cohort was mainly driven by the low positive percentage agreement of only 50.8% (118 SP142 IC ≥ 1% and 60 of these were also 22C3 CPS ≥ 10) where SP142 IC ≥ 1% expression was much more frequent than 22C3 CPS ≥ 10 and where 22C3 CPS 10 was not able to identify almost half (49.2%) of tumors that scored positive with SP142. Conversely, SP142 IC+ failed to identify 4.8% of tumors that scored positive with 22C3 CPS 10. We found better concordance rate of 81.5% (kappa value 0.68) when comparing SP142 IC ≥ 1% and 22C3 CPS ≥ 1, in line with two previously published studies [21, 25], though higher than reported by the IMpassion 130 sub-study of 63.5% [23]. The 22C3 CPS 1 scoring was not able to identify 15.3% of tumors that scored positive with SP142 and, on the other hand, SP142 was not able to identify 20.0% of tumors that scored positive with 22C3 CPS 1. We observed the best concordance rate of 86.6% between the two assays using the IC+ scoring for both (kappa value 0.73), which was in line with some previous results [25, 27, 28], but better than reported in the IMpassion 130 sub-study of 68.8% [23]. Our findings deviating from the IMpassion 130 sub-study might be explained by the lower rate of 22C3 CPS 1 and 22C3 IC+ positivity in our study, which in turn led to a substantially better negative percentage agreement in our cohort, resulting in a higher concordance rate.

PD-L1 (*CD274*) gene expression (mRNA) showed a strong positive association with all the IHC scorings of PD-L1 expression, irrespective of antibody and cut-off. PD-L1 gene expression could not explain the difference between SP142 and 22C3 CPS since both discordant groups (i.e. 22C3 CPS < 10 and SP142 IC ≥ 1% or 22C3 CPS ≥ 10 and SP142 < 1%) had similar PD-L1 gene expression levels.

We found that PD-L1 expression was positively associated with TIL abundance, NHG, Ki-67 level and histological medullary features. We also investigated the prognostic value of the different PD-L1 IHC scorings and found that PD-L1 expression when evaluated with SP142 IC+ and 22C3 CPS had a significant protective effect in patients that received (neo)adjuvant CT. However, PD-L1 status was not independently prognostic in multivariable regression analyses when adjusting for TIL abundance and other traditional prognostic features, where only TILs had an independent effect on outcome. Of the four different PD-L1 scorings and the three clinical endpoints, the prognostic impact of PD-L1 was strongest for 22C3 CPS ≥ 10 and DRFI. When dividing the CT-subgroup that had 22C3 CPS < 10 into SP142 IC positive and SP142 negative, we found that the SP142 status did not add any further prognostic value regarding DRFI if information for PD-L1 status with 22C3 is available. Keep in mind though that SP142 is relevant in predicting response to atezolizumab in the metastatic setting [6, 23]. It has previously been suggested that 22C3 is a better prognostic marker than SP142 in primary breast cancer patients [38] and our results suggest that 22C3 CPS at a threshold of 10 gives a better division into DRFI prognostic groups than SP142 IC+ in early-stage TNBC.

We chose to perform outcome analyses separately in the CT-cohort and the non-CT-cohort for several reasons. Older age and comorbidity (the primary reasons why (neo)adjuvant CT was not administered in the non-CT cohort), and thereby non-breast cancer related deaths in the non-CT-cohort, are competing risk factors regarding breast cancer specific events and diluting the OS results and, in part, the IDFS analyses. Moreover, TIL abundance and PD-L1 expression, both of which were lower in the non-CT-cohort than in the CT-cohort, are known to be positively associated with CT-response and prognosis in early TNBC [9, 10, 14, 33, 39, 40]. This in turn might partly explain why the prognostic impact of TILs and PD-L1 status was weaker than in the CT-cohort and not significant.

The population-based cohort is the main strength of our study, thus representing PD-L1 and TIL status in an early-stage TNBC population. A weakness is the small tissue cores in the TMA, potentially leading to inaccurate evaluations of PD-L1 expression due to intra-tumoral PD-L1 heterogeneity when compared to scoring on histological whole sections [21, 41–45]. Interestingly, neoadjuvant CT in TNBC is administered more frequently and becoming a standard of care compared to adjuvant CT. The evaluation of PD-L1 would be performed on core needle biopsies instead of whole sections in these patients, as it is often the case for metastatic lesions [46]. Core needle biopsy is more comparable with TMA in terms of size than whole section slides, and this aspect needs to be taken into consideration in the clinical setting when choosing thresholds for PD-L1 expression. Another caveat of our study is that we scored PD-L1 in primary TNBC tumors which have been found in a meta-analysis to differ from PD-L1 expression in metastatic lesions [47]. We have explored the analytical concordance of the SP142 and 22C3 assays. Unfortunately we cannot explore the predictive value of the interchangeability these assays due to the retrospective, non-randomized nature of our study where the patients did not receive immune checkpoint blockade. Further studies addressing that issue are warranted.

In summary, the PD-L1 IHC staining concordance between the clinically validated scoring algorithms for SP142 (IC ≥ 1%) and 22C3 (CPS ≥ 10) was impaired in our early-stage TNBC cohort. The concordance was better when evaluated with 22C3 CPS ≥ 1 or the same IC+ scoring method for both assays. The SP142 assay is better at identifying 22C3 positive tumors than the 22C3 assay is at identifying SP142 positive tumors. PD-L1 expression was of positive prognostic value in patients treated with (neo)adjuvant CT where it was strongest for DRFI and 22C3 CPS ≥ 10. However, PD-L1 status was not independently prognostic when adjusting for TIL abundance in multivariable analyses. Our findings suggest that these two antibody assays, with their respective clinically established scoring method and cut-offs, detect partially non-overlapping subpopulations of TNBC patients in the early-stage setting and are not substitutable with one another regarding PD-L1 detection and prognostic value. Further studies are warranted to investigate the predictive value of the interchangeability of these assays.

### Supplementary Information

**Additional file 1: Table S1.** Showing inter-core and interobserver PD-L1 concordances in the overall cohort

**Additional file 2: Table S2.** Showing clinicopathological features in the CT-cohort in relation to 22C3 CPS 1 and 22C3 IC PD-L1 status

**Additional file 3: Table S3.** Showing clinicopathological features in the non-CT-cohort in relation to PD-L1 status

**Additional file 4: Fig. S1.** Demonstrating Kaplan Meier estimates according to 22C3 CPS 1 and 22C3 IC status in the CT-cohort

**Additional file 5: Table S4.** Containing results from univariable regression analyses in the non-CT-cohort

**Additional file 6: Fig. S2.** Demonstrating Kaplan Meier estimates according to PD-L1 status in the non-CT-cohort

**Additional file 7: Table S5.** Containing clinicopathological features, follow-up data, PD-L1 and TIL scores used in our study

## Data Availability

The datasets used and/or analyzed during the current study are available from the corresponding author on reasonable request (clinicopathological features, PD-L1 and TIL scoring also available in Additional file 7: Table S5).
