# Supplementary material for: Comparison of SP142 and 22C3 PD-L1 assays in a population-based cohort of triple-negative breast cancer patients in the context of their clinically established scoring algorithms
Source: Breast Cancer Res. 2023 Oct 10;25:123. doi: 10.1186/s13058-023-01724-2 (PMC10566164; doi:10.1186/s13058-023-01724-2)
Supplement: Supplementary file 1 — Additional file 1: Table S1. Showing inter-core and interobserver PD-L1 concordances in the overall cohort [file 13058_2023_1724_MOESM1_ESM.docx]

| **Table S1**. Inter-core and interobserver concordances and kappa values in the overall cohort (N=232). | | | | | |
| --- | --- | --- | --- | --- | --- |
|  | | | | | |
| **Inter-core concordance, observer 1** |  |  |  |  |  |
|  | SP142 IC <1% | SP142 IC≥1% | Total | Concordance rate | Kappa |
| SP142 IC <1% | 114 | 13 | 127 |  |  |
| SP142 IC≥1% | 27 | 75 | 102 |  |  |
| Total | 141 | 88 | 229 | 82,5% | 0,64 |
|  |  |  |  |  |  |
| **Inter-core concordance, observer 2** |  |  |  |  |  |
|  | SP142 IC <1% | SP142 IC≥1% | Total | Concordance rate | Kappa |
| SP142 IC <1% | 115 | 17 | 132 |  |  |
| SP142 IC≥1% | 32 | 65 | 97 |  |  |
| Total | 147 | 82 | 229 | 78,6% | 0,55 |
|  |  |  |  |  |  |
| **Inter-core concordance,observer 2/observer 3** |  |  |  |  |  |
|  | 22C3 CPS <10 | 22C3 CPS ≥10 | Total | Concordance rate | Kappa |
| 22C3 CPS <10 | 0 | 11 | 11 |  |  |
| 22C3 CPS ≥10 | 10 | 41 | 51 |  |  |
| Total | 10 | 52 | 62 | 66,3% | <0,20 |
|  |  |  |  |  |  |
|  | 22C3 CPS <1 | 22C3 CPS ≥1 | Total | Concordance rate | Kappa |
| 22C3 CPS <1 | 101 | 10 | 111 |  |  |
| 22C3 CPS ≥1 | 20 | 91 | 111 |  |  |
| Total | 121 | 101 | 222 | 86,5% | 0,73 |
|  |  |  |  |  |  |
|  | 22C3 IC<1% | 22C3 IC≥1% | Total | Concordance rate | Kappa |
| 22C3 IC <1% | 127 | 17 | 144 |  |  |
| 22C3 IC ≥1% | 17 | 61 | 78 |  |  |
| Total | 144 | 78 | 222 | 84,7% | 0,66 |
|  |  |  |  |  |  |
| **Interobserver concordance (observer 1 and 2)** |  |  |  |  |  |
|  | SP142 IC <1% | SP142 IC≥1% | Total | Concordance rate | Kappa |
| SP142 IC <1% | 112 | 5 | 117 |  |  |
| SP142 IC≥1% | 6 | 109 | 115 |  |  |
| Total | 118 | 114 | 232 | 95,3% | 0,91 |

For the SP142 staining, one of the two TMA cores for three tumors was unevaluable/defected. For the 22C3 staining, one of the two TMA cores for ten tumors was unevaluable/defected.
