# Supplementary material for: Comparison of SP142 and 22C3 PD-L1 assays in a population-based cohort of triple-negative breast cancer patients in the context of their clinically established scoring algorithms
Source: Breast Cancer Res. 2023 Oct 10;25:123. doi: 10.1186/s13058-023-01724-2 (PMC10566164; doi:10.1186/s13058-023-01724-2)
Supplement: Supplementary file 2 — Additional file 2: Table S2. Showing clinicopathological features in the CT-cohort in relation to 22C3 CPS 1 and 22C3 IC PD-L1 status [file 13058_2023_1724_MOESM2_ESM.docx]

| Table S2. Clinicopathological features in the CT-cohort in relation to 22C3 CPS 1 and 22C3 IC PD-L1 status. | | | | | | |
| --- | --- | --- | --- | --- | --- | --- |
|  | **22C3 CPS 1 cut-off** | |  | **22C3 IC 1% cut-off** | |  |
| n (%) | **CPS <1**  N=68 (41.0%) | **CPS ≥1**  N=98 (59.0%) | **p-value** | **IC <1%**  N=91 (54.8%) | **IC ≥1%**  N=75 (45.2%) | **p-value** |
| Age at diagnosis, years |  |  |  |  |  |  |
| Median (range) | 58.5 (26-74) | 53.5 (28-76) | 0.123 | 58 (26-75) | 54 (28-76) | 0.317 |
| <50 y | 18 (26.5) | 33 (33.7) | 0.393 | 27 (29.7) | 24 (32.0) | 0.866 |
| ≥50 y | 50 (73.5) | 65 (66.3) |  | 64 (70.3) | 51 (68.0) |  |
| Tumor size |  |  |  |  |  |  |
| ≤20 mm | 32 (47.1) | 51 (52.0) | 0.742 | 43 (47.3) | 40 (53.3) | 0.872 |
| >20 mm | 30 (44.1) | 41 (41.8) |  | 38 (41.8) | 33 (44.0) |  |
| Unknown | 6 (8.8) | 6 (6.1) |  | 10 (11.0) | 2 (2.7) |  |
| Lymph node status |  |  |  |  |  |  |
| N0 | 41 (60.3) | 59 (60.2) | 1.000 | 53 (58.2) | 47 (62.7) | 0.633 |
| N+ | 27 (39.7) | 39 (39.8) |  | 38 (41.8) | 28 (37.3) |  |
| Histologic grade |  |  |  |  |  |  |
| 1 | 0 | 0 |  | 0 | 0 |  |
| 2 | 12 (17.6) | 2 (2.0) | <0.001 | 12 (13.2) | 2 (2.7) | 0.012 |
| 3 | 53 (77.9) | 93 (94.9) |  | 74 (81.3) | 72 (96.0) |  |
| Unknown | 3 (4.4) | 3 (3.1) |  | 5 (5.5) | 1 (1.3) |  |
| Ki-67 |  |  |  |  |  |  |
| ≤30% | 15 (22.1) | 7 (7.1) | 0.009 | 18 (19.8) | 4 (5.3) | 0.006 |
| >30% | 52 (76.5) | 91 (92.9) |  | 72 (79.1) | 71 (94.7) |  |
| Unknown | 1 (1.5) | 0 |  | 1 (1.1) | 0 |  |
| Histological type |  |  |  |  |  |  |
| Invasive ductal carcinoma | 55 (80.9) | 77 (78.6) | 0.002 | 74 (81.3) | 58 (77.3) | 0.002 |
| Medullary features | 1 (1.5) | 15 (15.3) |  | 3 (3.3) | 13 (17.3) |  |
| Other | 12 (17.6) | 6 (6.1) |  | 14 (15.4) | 4 (5.3) |  |
| TIL abundance |  |  |  |  |  |  |
| Median, % (range) | 10 (0-50) | 40 (1-90) | <0.001 | 10 (0-60) | 40 (5-90) | <0.001 |
| <30% | 61 (89.7) | 33 (33.7) | <0.001 | 73 (80.2) | 21 (28.0) | <0.001 |
| ≥30% | 7 (10.3) | 64 (65.3) |  | 17 (18.7) | 54 (72.0) |  |
| Unknown | 0 | 1 (1.0) |  | 1 (1.1) | 0 |  |
