# Supplementary material for: Comparison of SP142 and 22C3 PD-L1 assays in a population-based cohort of triple-negative breast cancer patients in the context of their clinically established scoring algorithms
Source: Breast Cancer Res. 2023 Oct 10;25:123. doi: 10.1186/s13058-023-01724-2 (PMC10566164; doi:10.1186/s13058-023-01724-2)
Supplement: Supplementary file 3 — Additional file 3: Table S3. Showing clinicopathological features in the non-CT-cohort in relation to PD-L1 status [file 13058_2023_1724_MOESM3_ESM.docx]

| Table S3. Clinicopathological features in the non-CT-cohort (N=66) in relation to PD-L1 status. | | | | | | |
| --- | --- | --- | --- | --- | --- | --- |
|  | **SP142 IC 1% cut-off** | |  | **22C3 CPS 10 cut-off** | |  |
| n (%) | **IC <1%**  N=42 (63.6%) | **IC ≥1%**  N=24 (36.4%) | **p-value** | **CPS <10**  N=50 (75.8%) | **CPS ≥10**  N=16 (24.2%) | **p-value** |
| Age at diagnosis, years |  |  |  |  |  |  |
| Median (range) | 80.5 (47-91) | 80 (38-90) | 0.664 | 80 (38-91) | 80 (46-91) | 0.887 |
| <50 y | 1 (2.4) | 2 (8.3) | 0.548 | 2 (4.0) | 1 (6.3) | 1.000 |
| ≥50 y | 41 (97.6) | 22 (91.7) |  | 48 (96.0) | 15 (93.8) |  |
| Tumor size |  |  |  |  |  |  |
| ≤20 mm | 20 (47.6) | 10 (41.7) | 0.798 | 24 (48.0) | 6 (37.5) | 0.569 |
| >20 mm | 22 (52.4) | 14 (58.3) |  | 26 (52.0) | 10 (62.5) |  |
| Lymph node status |  |  |  |  |  |  |
| N0 | 28 (66.7) | 17 (70.8) | 1.000 | 34 (68.0) | 11 (68.8) | 1.000 |
| N+ | 13 (31.0) | 7 (29.2) |  | 15 (30.0) | 5 (31.3) |  |
| Unknown | 1 (2.4) | 0 |  | 1 (2.0) | 0 |  |
| Histologic grade |  |  |  |  |  |  |
| 1 | 0 | 0 |  | 0 | 0 |  |
| 2 | 13 (31.0) | 3 (12.5) | 0.137 | 13 (26.0) | 3 (18.8) | 0.742 |
| 3 | 29 (69.0) | 21 (87.5) |  | 37 (74.0) | 13 (81.3) |  |
| Ki-67 |  |  |  |  |  |  |
| ≤30% | 13 (31.0) | 6 (25.0) | 0.778 | 16 (32.0) | 3 (18.8) | 0.357 |
| >30% | 28 (66.7) | 18 (75.0) |  | 33 (66.0) | 13 (81.3) |  |
| Unknown | 1 (2.4) | 0 |  | 1 (2.0) | 0 |  |
| Histological type |  |  |  |  |  |  |
| Invasive ductal carcinoma | 30 (71.4) | 21 (87.5) | 0.222 | 37 (74.0) | 14 (87.5) | 0.326 |
| Medullary features | 0 | 0 |  | 0 | 0 |  |
| Other | 12 (28.6) | 3 (12.5) |  | 13 (26.0) | 2 (12.5) |  |
| TIL abundance |  |  |  |  |  |  |
| Median, % (range) | 10 (0-50) | 35 (0-100) | <0.001 | 10 (0-70) | 30 (5-100) | 0.006 |
| <30% | 36 (85.7) | 9 (37.5) | <0.001 | 38 (76.0) | 7 (43.8) | 0.026 |
| ≥30% | 5 (11.9) | 15 (62.5) |  | 11(22.0 | 9 (56.3) |  |
| Unknown | 1 (2.4) | 0 |  | 1 (2.0) | 0 |  |
|  |  |  |  |  |  |  |
|  | **22C3 CPS 1 cut-off** | |  | **22C3 IC 1% cut-off** | |  |
|  | **CPS <1**  N=39 (59.1%) | **CPS ≥1**  N=27 (40.9%) | **p-value** | **IC <1%**  N=44 (66.7%) | **IC ≥1%**  N=22 (33.3%) | **p-value** |
| Age at diagnosis, years |  |  |  |  |  |  |
| Median (range) | 79 (47-91) | 80 (38-91) | 0.932 | 79.5 (47-91) | 80 (38-90) | 0.913 |
| <50 y | 1 (2.6) | 2 (7.4) | 0.563 | 1 (2.3) | 2 (9.1) | 0.256 |
| ≥50 y | 38 (97.4) | 25 (92.6) |  | 43 (97.7) | 20 (90.9) |  |
| Tumor size |  |  |  |  |  |  |
| ≤20 mm | 20 (51.3) | 10 (37.0) | 0.318 | 22 (50.0) | 8 (36.4) | 0.432 |
| >20 mm | 19 (48.7) | 17 (63.0) |  | 22 (50.0) | 14 (63.6) |  |
| Lymph node status |  |  |  |  |  |  |
| N0 | 24 (61.5) | 21 (77.8) | 0.278 | 29 (65.9) | 16 (72.7) | 0.780 |
| N+ | 14 (35.9) | 6 (22.2) |  | 14 (31.8) | 6 (27.3) |  |
| Unknown | 1 (2.6) | 0 |  | 1 (2.3) | 0 |  |
| Histologic grade |  |  |  |  |  |  |
| 1 | 0 | 0 |  | 0 | 0 |  |
| 2 | 13 (33.3) | 3 (11.1) | 0.045 | 14 (31.8) | 2 (9.1) | 0.066 |
| 3 | 26 (66.7) | 24 (88.9) |  | 30 (68.2) | 20 (90.9) |  |
| Ki-67 |  |  |  |  |  |  |
| ≤30% | 15 (38.5) | 4 (14.8) | 0.051 | 15 (34.1) | 4 (18.2) | 0.249 |
| >30% | 23 (59.0) | 23 (85.2) |  | 28 (63.6) | 18 (81.8) |  |
| Unknown | 1 (2.6) | 0 |  | 1 (2.3) | 0 |  |
| Histological type |  |  |  |  |  |  |
| Invasive ductal carcinoma | 27 (69.2) | 24 (88.9) | 0.078 | 31 (70.5) | 20 (90.9) | 0.071 |
| Medullary features | 0 | 0 |  | 0 | 0 |  |
| Other | 12 (30.8) | 3 (11.1) |  | 13 (29.5) | 2 (9.1) |  |
| TIL abundance |  |  |  |  |  |  |
| Median, % (range) | 10 (0-50) | 30 (5-100) | <0.001 | 10 (0-60) | 35 (5-100) | <0.001 |
| <30% | 34 (87.2) | 11 (3.7) | <0.001 | 37 (84.1) | 8 (36.4) | <0.001 |
| ≥30% | 4 (10.3) | 16 (59.3) |  | 6 (13.6) | 14 (63.6) |  |
| Unknown | 1 (2.6) | 0 |  | 1 (2.3) | 0 |  |
