# Supplementary material for: Comparison of SP142 and 22C3 PD-L1 assays in a population-based cohort of triple-negative breast cancer patients in the context of their clinically established scoring algorithms
Source: Breast Cancer Res. 2023 Oct 10;25:123. doi: 10.1186/s13058-023-01724-2 (PMC10566164; doi:10.1186/s13058-023-01724-2)
Supplement: Supplementary file 5 — Additional file 5: Table S4. Containing results from univariable regression analyses in the non-CT-cohort [file 13058_2023_1724_MOESM5_ESM.docx]

| Table S4. Univariable regression analyses in the non-CT-cohort (n=66). | | | | | | |
| --- | --- | --- | --- | --- | --- | --- |
|  | **IDFS**  (events=27) |  | **OS**  (events=21) |  | **DRFI**  (events=11) |  |
|  | **HR (95% CI)** | **p-value** | **HR (95% CI)** | **p-value** | **HR (95% CI)** | **p-value** |
| Age at diagnosis |  |  |  |  |  |  |
| Continuous | 1.05 (1.00-1.11) | 0.043 | 1.07 (1.01-1.14) | 0.027 | 1.04 (0.97-1.12) | 0.253 |
| Tumor size |  |  |  |  |  |  |
| ≤20mm | 1.00 |  | 1.00 |  | 1.00 |  |
| >20mm | 2.41 (1.08-5.39) | 0.033 | 2.54 (1.02-6.32) | 0.045 | 4.63 (1.00-21.53) | 0.051 |
| Lymph node status |  |  |  |  |  |  |
| N0 | 1.00 |  | 1.00 |  | 1.00 |  |
| N+ | 2.05 (0.93-4.54) | 0.076 | 1.74 (0.70-4.32) | 0.234 | 8.06 (2.13-30.59) | 0.002 |
| Histologic grade |  |  |  |  |  |  |
| 2 | 1.00 |  | 1.00 |  | 1.00 |  |
| 3 | 1.46 (0.58-3.63) | 0.421 | 1.96 (0.65-5.88) | 0.231 | 0.71 (0.21-2.42) | 0.581 |
| Ki67 |  |  |  |  |  |  |
| ≤30% | 1.00 |  | 1.00 |  | 1.00 |  |
| >30% | 1.45 (0.60-3.49) | 0.411 | 1.96 (0.71-5.46) | 0.196 | 0.61 (0.19-2.01) | 0.420 |
| TIL abundance |  |  |  |  |  |  |
| <30% | 1.00 |  | 1.00 |  | 1.00 |  |
| ≥30% | 0.91 (0.40-2.10) | 0.825 | 0.90 (0.34-2.33) | 0.821 | 1.34 (0.39-4.58) | 0.642 |
| SP142 IC+ |  |  |  |  |  |  |
| <1% | 1.00 |  | 1.00 |  | 1.00 |  |
| ≥1% | 0.55 (0.23-1.29) | 0.167 | 0.80 (0.32-1.99) | 0.637 | 0.70 (0.19-2.66) | 0.605 |
| 22C3 CPS 10 |  |  |  |  |  |  |
| <10 | 1.00 |  | 1.00 |  | 1.00 |  |
| ≥10 | 0.65 (0.24-1.71) | 0.377 | 0.91 (0.33-2.49) | 0.856 | 0.64 (0.14-2.94) | 0.562 |
| 22C3 CPS 1 |  |  |  |  |  |  |
| <1 | 1.00 |  | 1.00 |  | 1.00 |  |
| ≥1 | 0.55 (0.24-1.25) | 0.154 | 0.84 (0.35-2.04) | 0.704 | 0.56 (0.15-2.12) | 0.395 |
| 22C3 IC+ |  |  |  |  |  |  |
| <1% | 1.00 |  | 1.00 |  | 1.00 |  |
| ≥1% | 0.53 (0.21-1.35) | 0.171 | 0.80 (0.31-2.07) | 0.648 | 0.77 (0.20-2.90) | 0.699 |
