# Supplementary material for: Comparison of SP142 and 22C3 PD-L1 assays in a population-based cohort of triple-negative breast cancer patients in the context of their clinically established scoring algorithms
Source: Breast Cancer Res. 2023 Oct 10;25:123. doi: 10.1186/s13058-023-01724-2 (PMC10566164; doi:10.1186/s13058-023-01724-2)
Supplement: Supplementary file 6 — Additional file 6: Fig. S2. Demonstrating Kaplan Meier estimates according to PD-L1 status in the non-CT-cohort [file 13058_2023_1724_MOESM6_ESM.pdf]

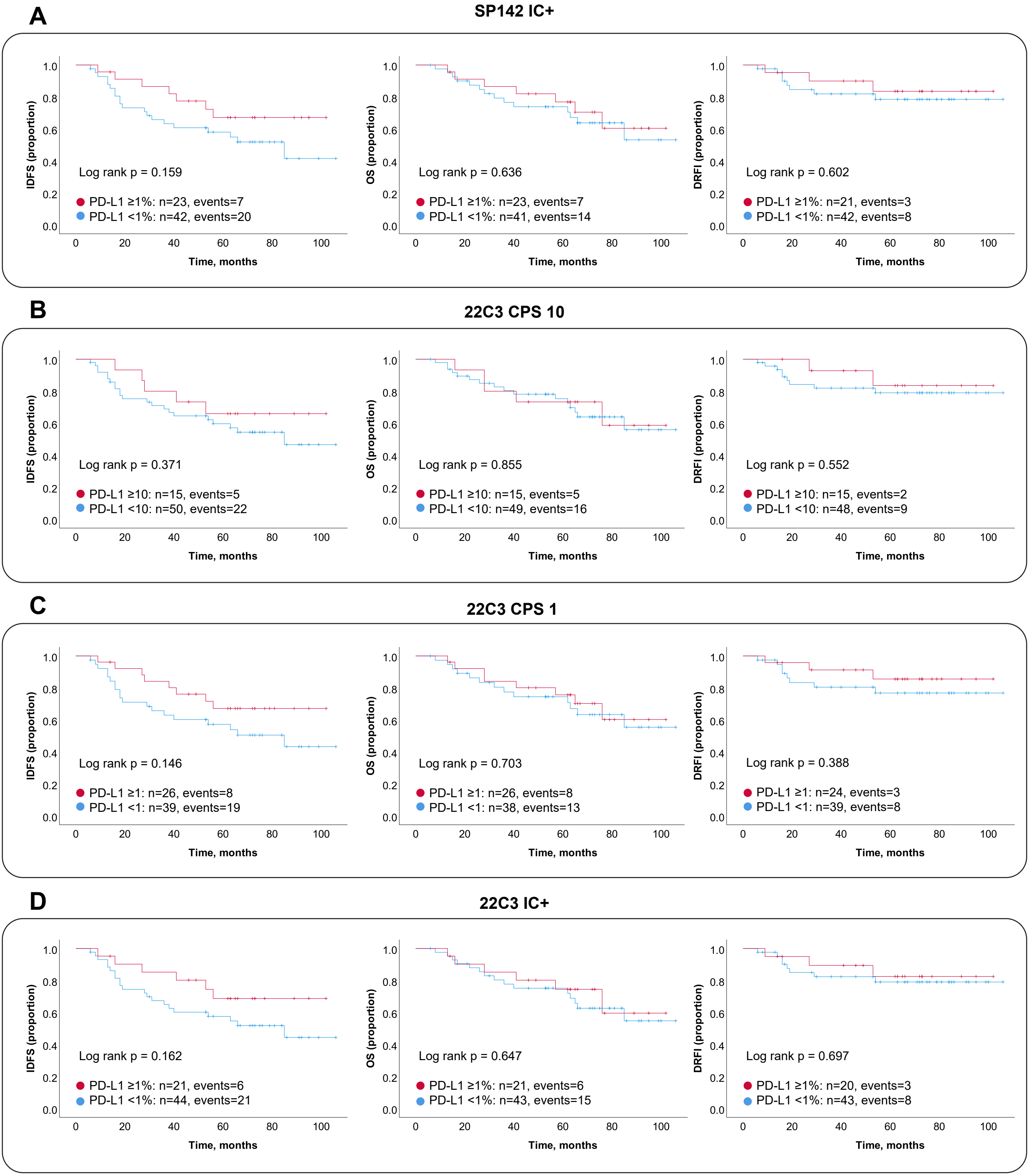

Fig. S2 Kaplan Meier survival analyses in the cohort not receiving (neo)adjuvant chemotherapy. Invasive disease-free survival (IDFS), overall survival (OS) and distant relapse-free interval (DRFI) according to PD-L1 SP142 immune cell staining (IC+) in panel **(A)**, in panel **(B)** for 22C3 combined positive score (CPS) at a threshold of 10, in (**C)** according to 22C3 CPS at a threshold of 1 and in **(D)** for 22C3 IC+.
